# IUC24406-81 ARTA-ATTACK study: assessment of sarcopenia, frailty, and neurocognitive impairment in patients with prostate cancer treated with androgen receptor-targeted agents: a prospective multicenter observational cohort study

**DOI:** 10.1093/oncolo/oyaf248.029

**Published:** 2025-08-29

**Authors:** G Banna, A Maniam, P Rescigno, A Reid, S Crabb, M Maffezzoli, B Smalley

**Affiliations:** Portsmouth Hospital University Trust, United Kingdom; Portsmouth Hospital University Trust, United Kingdom; Department Translational and Clinical Research Institute, Newcastle University, United Kingdom; The Royal Marsden NHS Foundation Trust, United Kingdom; University Hospital Southampton NHS Foundation Trust, United Kingdom; Portsmouth Hospitals University NHS Trust, United Kingdom; Portsmouth Hospitals University NHS Trust, United Kingdom

## Abstract

**Background:**

Treatment of prostate cancer has evolved significantly over the past decade with the introduction of androgen ­receptor-­targeted agents (ARTAs) such as enzalutamide, abiraterone, apalutamide, and darolutamide. These agents have demonstrated improved overall survival and progression-free survival compared to androgen deprivation therapy (ADT) alone. However, their impact on functional status, frailty, and quality of life, especially in older patients, remains poorly characterized.

**Methods:**

Primary Objective: To evaluate changes in functional and neurocognitive status, frailty, and body composition in patients with hormone-sensitive prostate cancer treated with ARTAs over 30 months. Measures: Change in G8 geriatric screening score, SARC-F questionnaire, Muscle Mass, Mini-Cog, age-adjusted Charlson Comorbidity Index, ECOG performance status, Activities of Daily Living, Instrumental Activities of Daily Living, Nutritional Risk Index, Body fat percentage and QRISK3 cardiovascular risk from baseline to 30 months. Secondary Objectives: (1) To compare the impact of different ARTAs on functional and neurocognitive status, frailty, and body composition in patients with hormone-sensitive prostate cancer over 30 months. Measures: Change in the primary outcome measures for each ARTA type, incidence and severity of adverse events. Time to PSA progression and radiographic progression-free survival for each treatment group. (2) To develop and validate a simplified tool incorporating key items from validated scales assessing function, cognition, and frailty. Measures: Development and validation of a simplified tool incorporating key items from validated scales. Partnership with the ONCOassist platform to implement a simple tool for the capture of validated cognitive/frailty/sarcopenia measures. Impedance meter scales for body composition measurements. Radiological imaging at baseline and during follow-up, as per standard of care (MRI, PET-CT or CT scan)

**Baseline assessment:**

Comprehensive evaluation using the ONCOassist tool and bioimpedance analysis. Radiological imaging at baseline and analyzed using OsiriX MD (macOS) or sliceOmatic (other operating systems), to measure body fat and muscle mass.

**Results:**

Treatment period: Participants will receive their prescribed ARTA treatment as part of standard care. Follow-up assessments: Measures of primary and secondary outcomes at 3 and 6 months after baseline, then every 6 months to 30 months. Radiological imaging after baseline will be performed as per standard of care.

**Conclusions:**

This study will provide real-world evidence on how ARTAs impact functional status, frailty, cognition, and body composition in patients with hormone-sensitive prostate cancer. Findings will inform treatment choices, particularly in older or vulnerable populations. The development of a simplified screening tool may support routine assessment and more individualized care in clinical practice.

**Figure 1:**
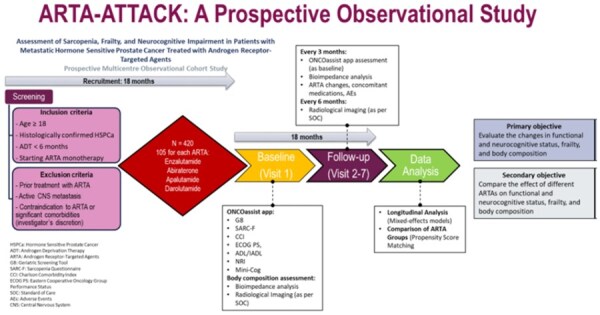
Summary of the ARTA-Attack trial protocol.

